# Progress or Precipice? – A Service Evaluation of a Specialist Eating Disorder Unit Serving Both Adolescents and Adults

**DOI:** 10.1192/bjo.2025.10484

**Published:** 2025-06-20

**Authors:** Giles Glass, Lara Harrison, Val Yeung

**Affiliations:** 1 Elysium Healthcare, Worcestershire, United Kingdom; 2 SupportED, West Midlands, United Kingdom

## Abstract

**Aims:** There is good qualitative evidence in the literature of the challenges of transition from Child and Adolescent Mental Health Services (CAMHS) to Adult services at aged 18 faced by young people, their families or carers and professionals. Eating disorders typically present in adolescents and persist into early adulthood with an average age of onset at around age 18. This population is therefore often faced with the challenge of transitioning between services during periods of treatment. Some community eating disorder teams in the UK have started to move towards an all-age model, however, inpatient services do not seem to have kept pace with this change.

**Methods:** A literature search using PubMed was conducted to identify any publications relating to the transition between CAMHS and adult services in eating disorder treatment. An evaluation of the service at Cotswold Spa Hospital was done, and a review of admissions and discharges in the last year. The evaluation aimed to identify, understand and assess the ability to transition from CAMHS to Adult services in an eating disorder inpatient setting.

**Results:** Cotswold Spa Hospital is a private provider of NHS commissioned inpatient eating disorder treatment. It offers both acute inpatient and day patient eating disorder treatment to CAMHS and adult patients with the same treating team at one site. This allows the potential for young people to transition from CAMHS to Adult services whilst undergoing inpatient or day patient treatment, without the need to move setting during this most crucial part of their recovery. It is one of very few settings in the UK where this is possible at present. There are 8 CAMHS beds and 4 adult beds on different floors of the hospital, and the day unit is in a separate building. In the last year (2024) four patients were admitted aged 17 and continued their treatment at Cotswold hospital beyond their 18th birthday. Without this service it is likely that their care would have been interrupted with an inpatient transfer.

**Conclusion:** Transition between CAMHS and Adult services at age 18 whilst undergoing inpatient eating disorder treatment presents numerous challenges. Service evaluation identified Cotswold Spa Hospital offers a rare approach which can avoid this disruption to recovery by continuing care in the same setting.

